# Electrical Conductivity as an Informative Factor of the Properties of Liposomal Systems with Naproxen Sodium for Transdermal Application

**DOI:** 10.3390/ma17225666

**Published:** 2024-11-20

**Authors:** Witold Musiał, Carla Caddeo, Alina Jankowska-Konsur, Giorgio Passiu, Tomasz Urbaniak, Maria Twarda, Adam Zalewski

**Affiliations:** 1 Department of Physical Chemistry and Biophysics, Wroclaw Medical University, Borowska 211A, 50-556 Wrocław, Poland; 2 Department of Life and Environmental Sciences, University of Cagliari, SS 554—Bivio per Sestu, 09042 Monserrato, Italy; caddeoc@unica.it (C.C.);; 3 Clinical Department of Oncodermatology, University Centre of General Dermatology and Oncodermatology, Wroclaw Medical University, Borowska 213, 50-556 Wrocław, Poland; alina.jankowska-konsur@umw.edu.pl (A.J.-K.);

**Keywords:** nonsteroidal anti-inflammatory drugs, transdermal drug delivery, dynamic viscosity, conductometry, non-ionic polymer, polymeric gel

## Abstract

Liposomal preparations play an important role as formulations for transdermal drug delivery; however, the electrical conductivity of these systems is sparingly evaluated. The aim of the study was to outline the range of the values of electrical conductivity values that may be recorded in the future pharmaceutical systems in the context of their viscosity. The electrical conductivity, measured by a conductivity probe of k = 1.0 cm^−1^, and the dynamic viscosity of liposomal and non-liposomal systems with naproxen sodium, embedded into a methylcellulose hydrophilic gel (0.25%), were compared with data from preparations without naproxen sodium in a range reflecting the naproxen sodium concentrations 0.1·10^−2^–9.5·10^−2^ mol/L. The specific conductivity covered a 1.5 μS·cm^−1^–5616.0 μS·cm^−1^ range, whereas the viscosity ranged from 0.9 to 9.4 mPa·s. The naproxen sodium highly influenced the electrical conductivity, whereas the dynamic viscosity was a moderate factor. The observed phenomena may be ascribed to the high mobility of sodium ions recruited from naproxen sodium and the relatively low concentrations of applied methylcellulose. The assembly of lecithin in liposomes may have lowered the specific conductivity of the systems with naproxen sodium. These measurements will be further developed for implementation as simple assays of the concentrations of active pharmaceutical ingredient in release experiments of preparations proposed for dermatological applications.

## 1. Introduction

Naproxen is a commonly used non-steroidal anti-inflammatory drug (NSAID) for managing pain and inflammation, particularly in chronic conditions like arthritis [1]. However, the oral administration of naproxen may result in gastrointestinal issues and other systemic side effects [1,2]. This has led to the exploration of transdermal drug delivery systems (TDDSs) as an important alternative, enabling localized drug delivery with minimized systemic exposure. Liposomes, which are nanocarriers made of lipid bilayers, have been extensively studied as effective carriers for enhancing naproxen’s transdermal delivery due to their ability to penetrate the skin’s outer barrier, the stratum corneum [2,3,4].

The liposomes’ lipid bilayer structure surrounding an aqueous core, which closely resembles that of biological membranes, allows them to entrap both hydrophilic and lipophilic compounds. This structure enhances the ability of liposomes to penetrate the skin’s outer barrier, the stratum corneum, thereby improving the delivery of drugs like naproxen to deeper layers of the skin [2,5]. According to a review by Kumar et al., liposomes enhance drug permeation and bioavailability, providing a sustained release profile that is beneficial for managing chronic inflammatory conditions requiring prolonged therapeutic effects [6].

The validity of the development of various liposomal structures for the enhancement of intradermal and transdermal transport was confirmed by numerous authors [5,7,8,9,10,11]. These advancements are supported by findings from Puglia et al., which demonstrate that liposomal formulations of naproxen-containing phosphatidylcholine and cholesterol show enhanced permeation through human skin compared to conventional formulations [2].

Combining liposomes with microneedle technology further enhances naproxen delivery by creating microchannels in the skin that facilitate deeper drug penetration. This combined approach not only enables the rapid onset of action but also ensures sustained release, making it suitable for acute flare-ups in chronic inflammatory conditions. The integration of microneedles with liposomal delivery systems has shown potential for improving bioavailability and therapeutic outcomes for transdermal NSAID delivery [7,12].

Overall, the use of liposomes, and other advanced lipid-based carriers, represents a significant advancement in the transdermal delivery of naproxen. These systems overcome the limitations of conventional liposomes by enhancing drug penetration, providing controlled release, and reducing systemic side effects. While clinical application remains challenging, the continued development of these technologies promises to improve the management of pain and inflammation for patients requiring long-term NSAID therapy [3].

The dissolution studies, realized in the process of the development of innovative drug carriers for controlled and targeted drug delivery, employ many analytical methods. However, the main hindrance of the applied methods often includes the necessity of the use of complicated and long procedures due to the high number of samples in one set. The application of a simple conductance sensor may shorten the procedure and enable an on-line visualization of the release profiles, which presently are usually combined after laborious proceeding. The evaluation of the level of hereby assessed naproxen sodium in preparations containing several components, and prepared in a specific way, e.g., as hydrogel preparations, needs the application of a specific and often extensive number of methods, including complicated devices [13]. Also, the thermodynamic activity of the systems, or thermodynamic activities of the active components, could be recorded in the evaluated systems [14]. One of the ways to reflect the concentration and transport of the components in preparations, and in assessed pharmaceutical systems, is the measurement of its electrical conductivity [15]. In our previous manuscripts, we presented a feasible method to obtain the levels of ferrous ions in the acceptor system, ions which originated from the assessed commercial drugs—tablets—and capsules of prolonged drug release [16]. More complicated systems include liposomal preparations, where the active pharmaceutical ingredient is implemented into vesicles composed of amphiphilic particles, deposited in hydrophilic, polymeric gel [17]. For some pharmaceutical applications, specific and highly specialized electrochemical sensors were constructed [18,19]. Thus, it is extremely interesting to obtain data on the conductivity of such systems and to identify the main sources of the signal in the measurements of the electrical conductivity of complex liposomal systems loaded with naproxen sodium and embedded in a polymer matrix.

The aim of the present study was to identify, and to outline, the range of electrical conductivity values that may be recorded in future pharmaceutical systems, which contain active pharmaceutical ingredients and other functional components important for the proper activity of the entire dosage form. This may lead to the elucidation of the relationship between conductivity, viscosity, and naproxen sodium concentration in such types of dosage forms. The study covered the electrical conductivity of liposomal system made of lecithin and naproxen sodium, immersed in the hydrophilic gel composed of methylcellulose.

## 2. Materials and Methods

### 2.1. Materials

Naproxen sodium (NS, (S)-(+)-2-(6-Methoxy-2-naphthyl)propionic sodium, CAS: 26159-34-2, conforming to USP standards (Sigma-Aldrich, Sternheim, Germany), soy lecithin (ECOSPA, Warszawa, Poland), methylcellulose (MC) of 4000 cP per 2% of aqueous solution at 20 °C (Sigma-Aldrich, Steinhelm, Germany), chloroform (Merck, Darmstadt, Germany), and deionized water from purification station (Wroclaw Medical University, Wrocław, Poland).

### 2.2. Preparation of Liposomes

Liposomes were prepared according to the quantities reported in Table 1. Soy lecithin was dissolved in chloroform in a vacuum flask, and the solvent was evaporated in a rotavapor (Chemland, Stargard, Poland) at 62 °C and 300 rpm. The formed film was sonificated (Polsonic, Warsaw, Poland) and dried for 1 h 30 min in a dryer (Chemland). Water or naproxen solution was added, and the dispersion was placed in a mechanical stirrer for 10 min at a speed of 250 rpm. The procedure was analogous to that performed in our former research [20]. Once the liposomes were obtained, they were extruded using a mini extruder (Avanti Polar Lipids 610020, Alabaster, AL, USA) with polycarbonate filters of 0.2 and 0.1 μm (Whatman^®^ Nuclepore™ Track-Etched Membranes, Merck KGaA, Darmstadt, Germany) using the method proposed i.a. by Luo et al. [21]. The obtained dispersions of liposomes were used in further experiments.

### 2.3. Preparation of Hydrophilic Gels and Physical Mixtures

The hydrophilic gels were obtained by mixing: (1) water and methylcellulose, (2) aqueous solution of naproxen sodium and methylcellulose, (3) aqueous dispersion lecithin, water, and MC, (4) aqueous dispersion of liposomes, water, and MC, and (5) aqueous dispersion of liposomes with naproxen sodium, water, and MC (Table 1). The hydrophilic gels were stabilized at 6 °C for 24 h to enable entire swelling of methylcellulose and removal of the air. The respective dilutions were prepared to enable evaluation of the samples concentration on the recorded properties of the analyzed systems. The physical mixtures were prepared similarly by mixing the components in proportions outlined in Table 1.

### 2.4. Measurements of the Conductivity

The conductivity meter with the respective conductivity probes (Elmetron conductivity meter CC-505 and conductivity probe EC-70 of k = 1.0 cm^−1^, Elmetron, Zabrze, Poland) was applied to measure the electric conductivity of prepared samples at 298 K. Consequently, the electrical conductivity of diluted samples was also analyzed to enable the observation of the influence of sample concentration on the conductivity values. The diluted samples were obtained via analytical dilution in *v*/*v* proportions: 1:2, 1:4, 1:8, 1:16, 1:32, and 1:64, according to Table 1’s column of evaluated dilutions of prepared formulations. The data were recorded directly from the conductivity meter in µs·cm^−1^, and every measurement was repeated five times. The molar conductivity ($\Lambda_{s}$, Equation (1)) and limiting molar conductivity ($\Lambda_{0}$, Equation (2)) were calculated using recorded data of specific conductivity ($\kappa_{s}$, Equation (3)) of evaluated systems in regard to the molar concentrations ($c_{mol,\;Nap}$) of naproxen sodium in an aqueous environment (A~60.2, B~0.229).

(1) $$\Lambda_{s}=\kappa_{s}\cdot \frac{1000}{c_{mol,\;Nap}}\left[\mu S\cdot cm^{2}\cdot {mol}^{-1}\right]$$

(2) $$\Lambda_{0}=\frac{\Lambda_{s}+A\sqrt{c_{mol,\;Nap}}}{1+B\sqrt{c_{mol,\;Nap}}}\left[\mu S\cdot cm^{2}\cdot {mol}^{-1}\right]$$

(3) $$\kappa_{s}\left[\mu S\cdot {cm}^{-1}\right]$$

### 2.5. Evaluation of the Viscosity of Assessed Samples

The Ostwald viscometer was applied in viscosity measurements. The flux times of the tested formulations and water, as the reference liquid, were assessed in five replicates at 25 °C. The hydrostatic balance (WX-001-0001 system with AS X2 PLUS balance, Radwag, Radom, Poland) was used to determine the densities of the formulations and density of the water as the standard fluid in five replicates. The dynamic viscosities of the systems (η) were calculated on the basis of the density of the sample ($\varrho_{s}$), the reference fluid ($\varrho_{0}$), and the respective flux times ($t_{s}$, $t_{0}$) using the relative viscosity ($\eta_{r}$, Equation (4)).

(4) $$\eta_{r}=\frac{\varrho_{s}t_{s}}{\varrho_{0}t_{0}}$$

### 2.6. Evaluation of Model Plots, Standard Deviation, and Determination Coefficients

The linear regression model (Equation (5)), exponential regression model (Equation (6)), power regression model (Equation (7)), and logarithmic regression model (Equation (8)) were applied to evaluate the most appropriate course of the functions describing the resulting relations presented in the Discussion section (Table 2). The respective determination coefficients for proposed models ($r^{2}$) and standard deviation (SD) for every measurement point were also calculated. The data presented on the graphs include the mean values calculated on the basis of respective measurements of conductivity and viscosity.

(5) y=ax+b

(6) $$y={ab}^{x}$$

(7) $$y=ax^{b}$$

(8) y=aln⁡x+b

## 3. Results

### 3.1. The Specific Conductivity

**Figure 1:**
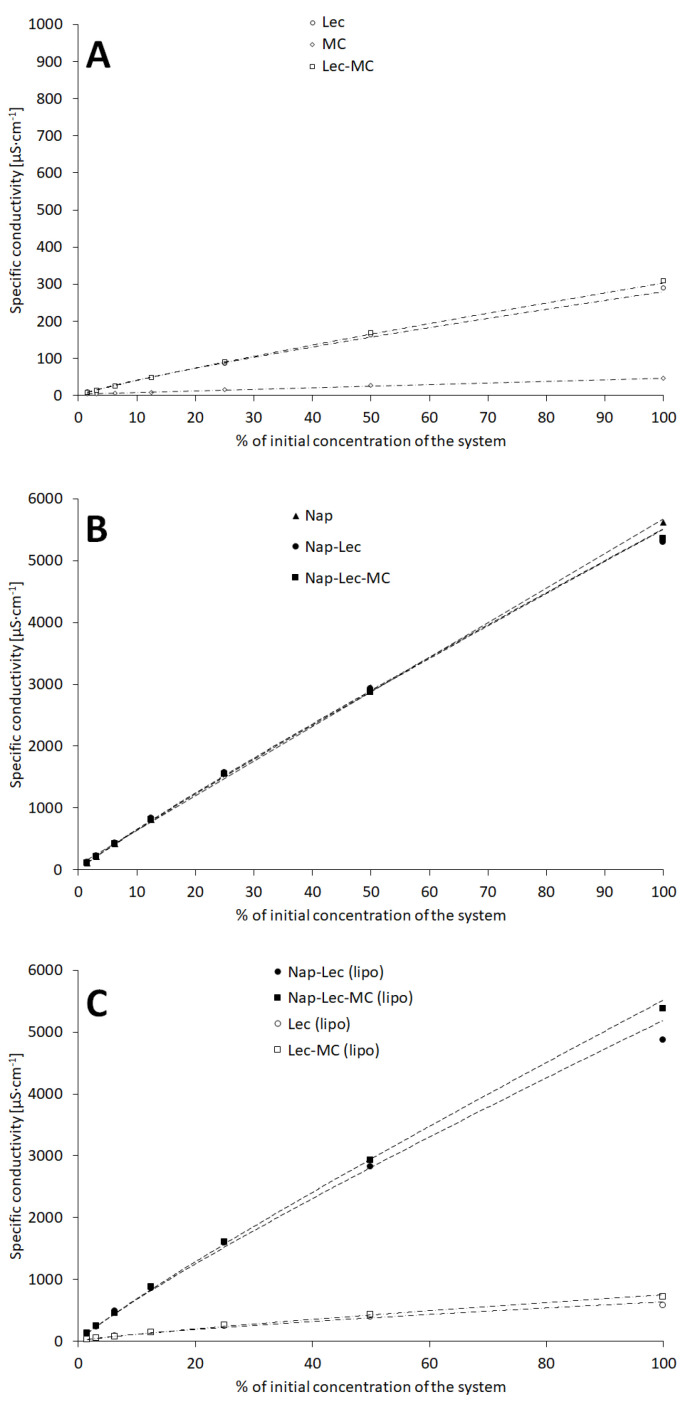
The influence of concentration on the recorded specific conductivity of evaluated systems: physical mixtures in absence of naproxen sodium (**A**), physical mixtures in the presence of naproxen sodium (**B**), and liposomal formulations with and without naproxen sodium (**C**). Composition details and abbreviations are outlined in Table 1.

The main measurements included the evaluation of the specific conductivity of the prepared systems (Figure 1A). The conductivity increased with the increase in the concentration of the system in all of the assessed samples.

However, the increase was rather small in the case of aqueous dispersions of evaluated components applied for the formation of liposomes (Figure 1A). The lowest registered electrical conductivity of methylcellulose dispersion was ca. 1.5 μS·cm^−1^ and did not exceed 50 μS·cm^−1^. The aqueous lecithin dispersion, as well as aqueous lecithin dispersion with methylcellulose, maintained a similar level of electrical conductivity in the range between 10.1 μS·cm^−1^ and 290.8 μS·cm^−1^ and 8.8 and 309 μS·cm^−1^, respectively. For comparison, the water conductivity was 1.2 μS·cm^−1^. The aqueous dispersions of lecithin and lecithin with methylcellulose, which contained naproxen sodium, had remarkably higher electrical conductivity (Figure 1B). The values ranged 114.0–5294.0 μS·cm^−1^ and 110.9–5352.0 μS·cm^−1^, respectively. The electrical conductivity of naproxen sodium dissolved in water in the parallel concentrations represented ranged between 106.7 and 5616.0 μS·cm^−1^. The conductivities of applied components, formed into dispersions that contained liposomes, were characterized by electrical conductivities similar to that recorded for physical mixtures of the used components; however, some differences were observed (Figure 1C). The electrical conductivities of the empty liposomes and the empty liposomes in methylcellulose matrix were in the range 26.8–575.9 μS·cm^−1^ and 23.1–718.2 μS·cm^−1^, respectively. Markedly higher values of electrical conductivity were observed for the liposomal preparation, containing naproxen sodium, as such or embedded in the methylcellulose hydrogel. The ranges of electrical conductivity were 124.6–4856.6 μS·cm^−1^ and 123.9–5381.2 μS·cm^−1^ respectively.

### 3.2. The Molar Conductivity

**Figure 2:**
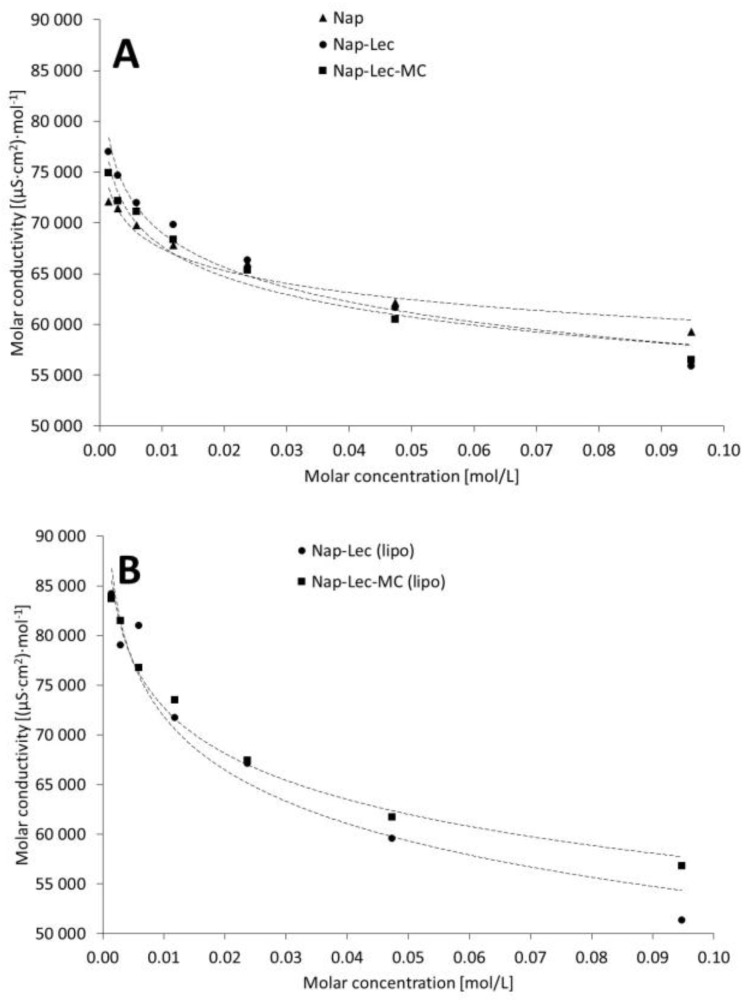
The influence of naproxen sodium molar concentration on the recorded molar conductivities of evaluated systems, calculated according to the API concentration in physical mixtures (**A**) and liposomal formulations (**B**). Composition details and abbreviations are outlined in Table 1.

The molar conductivity calculated in regard to the naproxen sodium varied between samples obtained on the basis of liposomal preparation and on the basis of mixtures of the components (Figure 2).

The recorded electrical conductivities of the aqueous solution of naproxen sodium, as well as of the physical mixtures with lecithin or with lecithin and methylcellulose, were in the range of 55,865.6–77,019.0 μS·cm^−1^. The same concentrations of components, although with formed liposomes, gave the broader range of conductivities of 51,344.9–84,164.4 μS·cm^−1^.

### 3.3. The Limiting Molar Conductivity

**Figure 3:**
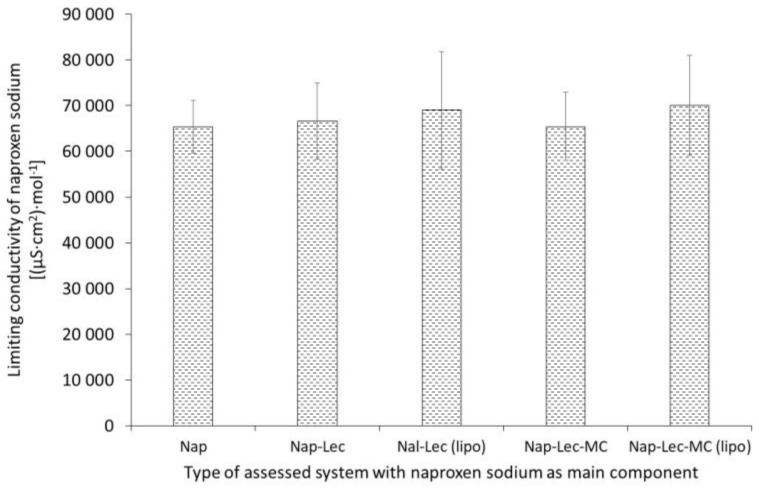
The limiting molar conductivity of naproxen sodium of evaluated systems. Composition details and abbreviations are outlined in Table 1.

Based on the data presented in Figure 3, there was no substantial difference between the calculated limiting molar conductivities of naproxen sodium in the studied systems.

### 3.4. Dynamic Viscosity

**Figure 4:**
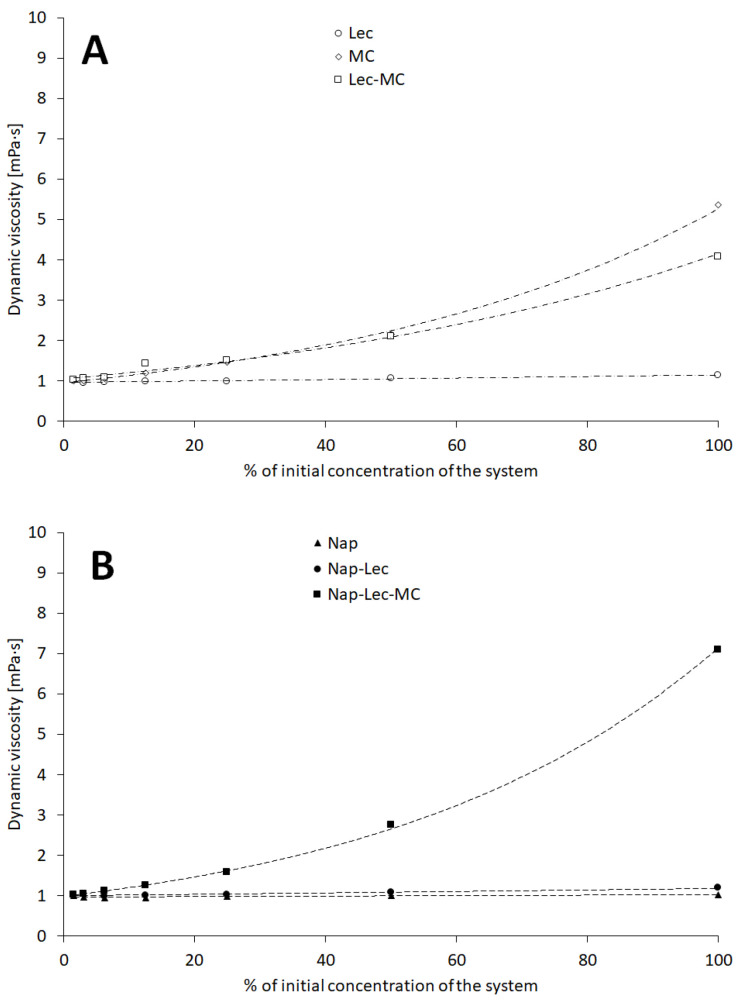

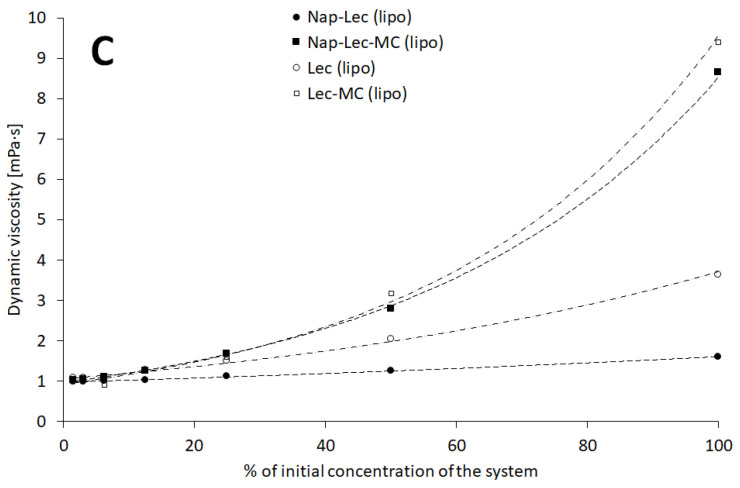
The influence of the concentration of the assessed system on the relative viscosity: the physical mixtures in absence of naproxen sodium (**A**), the physical mixtures in the presence of naproxen sodium (**B**), and the liposomal formulations with and without naproxen sodium (**C**). Composition details and abbreviations are outlined in Table 1.

The dynamic viscosities of the assessed systems in most cases increased with the concentration of the components (Figure 4).

The viscosities of the aqueous dispersions of the lecithin, methylcellulose, and aqueous dispersions range, respectively, as follows: 0.9–1.2 mPa·s, 1.0–5.4 mPa·s, and 1.0–4.1 mPa·s—Figure 4A. The physical mixtures of components, which reflected the compositions of liposomal formulations, exhibited a higher range of viscosities when methylcellulose was included—Figure 4B—and the maximal recorded dynamic viscosity was 7.09 mPa·s. A similar tendency was observed in the case of liposomal preparation—Figure 4C—where the maximal viscosity through the entire set of measurements was recorded in number 9.4 mPa·s.

The evaluated relations, including the dilution-specific conductivity of the systems ($\kappa_{s}=f\left(c_{\%ICS}\right)$, Equation (9)), the naproxen molar concentrations–molar conductivity of the systems ($\Lambda_{s}=f\left(c_{mol,\;Nap}\right)$, Equation (10)), and the dilution-dynamic viscosity of the systems ($\eta =f\left(c_{\%ICS}\right)$, Equation (11)), were fitted to four model functions described in the Methods section. Table 2 presents the resulting model functions, which were characterized by highest determination coefficients.

The first relation (Equation (9)) most often was best fitted to the power function, and the second relation (Equation (10)) in all cases fitted best to the logarithmic function. The best regression for the dependence between the dilution and dynamic viscosity of the systems (Equation (11)) was calculated as an exponential function.

## 4. Discussion

### 4.1. Specific Conductivity and Equations

The naproxen sodium was the main type of molecule that significantly influenced the level of specific conductivity in the evaluated systems, mainly due to the relatively high conductivity of the sodium ion, as well as the associated naproxen anion. The specific conductivity of systems with sodium naproxen was ca. 10-fold higher when compared to systems without the API (Figure 1). The difference of similar magnitude was observed both in liposomal and non-liposomal systems. The presence of lecithin was another factor, which added to the increase in specific conductivity. However, the influence of lecithin was much lower, comparing to naproxen sodium, which may be ascribed to the rather low mobility of the respective, variable ions included in the composition of the commercial lecithin used for the formation of liposomes in the pharmaceutical and cosmetic industry [22,23]. Additionally, the surface phenomena often involve the alternation of the electrical conductivity in systems containing surfactants and drugs [24,25,26]. The regression calculations indicate the power function as close to the plots representing the influence of the system concentration on the specific conductivity (Table 3, Equation (9)). Thus, there are some factors that limit the linear increase in recorded conductivity. This is in agreement with the theoretical considerations on the influence of ion concentration on the specific conductivity [27,28], although the influence in the recorded range is poorly expressed.

### 4.2. Molar Conductivity and Equations

Our recorded data of specific conductivities summarize the influence of all of the present ions on the conductivity phenomena in the sample; thus, some recalculations were performed to observe the influence of the naproxen sodium on the concentration–conductivity plots. The presented molar conductivities have some limitation as they were calculated according to the molar concentration of naproxen sodium in the system; however, they are still informative and may be useful to bring closer the phenomena of conductivity to the applicative field in pharmaceutical systems. The most interesting finding was the variability between the range of molar conductivity in the non-liposomal systems (Figure 2A) and liposomal systems (Figure 2B). Both the range and the slope (Table 3, Equation (10)) were higher in the case of liposomal preparations. The higher values of molar conductivities observed in liposomal systems may be ascribed to the higher dispersion of the lecithin due to the unification process of the liposomes, which could result in a higher number of molecularly dispersed lecithin particles. Another explanation may be applied to the samples of high concentrations where the presence of lecithin in the form of more densely packed liposomes may hinder the ion mobility. The section on the relations’ dynamic viscosity-specific conductivity also enables some insight into possible phenomena (Figure 4, Table 3). The observations would be evaluated in further studies.

### 4.3. Limiting Molar Conductivity

To fulfill the landscape of conductivity in the evaluated samples, we calculated the limiting molar conductivities. The limiting molar conductivity of the naproxen sodium in the aqueous solution obtained from the literature was between ca. 50 and 114 S·cm^2^·mol^−1^ in water at temperatures ranging 278.15–308.15 K, according to the bibliography [29]. In our research, it was established on the lower levels (Figure 3, Nap system), which may be ascribed to the variability in the environmental parameters and the calculative method. It should be emphasized that the same procedure was applied for all the systems, which ensures the possibility of a comparison of the assessed preparations. The limiting molar conductivities did not vary in the terms of statistical significance, as is proven in Figure 3; however, some tendency was observed, which was in agreement with the data from the molar conductivity of the assessed systems; slightly higher values were observed in the liposomal systems.

### 4.4. Dynamic Viscosity

The dynamic viscosity of the liposomal systems was strongly affected by the presence of the nonionic polymer methylcellulose (Figure 4A–C). The linear increase in the *w*/*w* concentration of the polymer resulted in an exponential increase in the viscosity of the systems (Table 2, Equation (11)), which is in agreement with the remarks of other researchers who studied both ionic [30] and non-ionic [31,32] polymer solutions. An interesting phenomenon was observed when the liposomal systems with and without naproxen sodium were compared—the viscosities of the systems loaded with naproxen sodium were remarkably higher in Figure 4C. As the only variable was the presence of naproxen sodium, it may be concluded that the active pharmaceutical ingredient altered the properties of the liposomal systems, which could be assisted by the applied soya lecithin formed as liposomal particles. The electrolytes often influence the properties of colloidal systems [33,34] due to the interactions between the polyelectrolyte chains’ functional groups and the small ions. However, in the considered systems, the only polymeric molecule was nonionic, and the interaction may be ascribed to another sort of interaction in quaternary mixture where the liposomes are present. One of the explanations may involve the possible physical bonding between the formed liposomes and the polymeric environment. The bonds in this case may be stronger than the interactions between the dispersed molecules of soya lecithin and the polymer in the physical mixtures, thus limiting the movement of the liquid layers during the viscosity measurements. The idea of the lecithin influencing the restructuring of the polymeric hydrogel network was detailed by Pang [35]. The similar, but not identical, behavior of the polymeric system in the presence of liposomes was described by Menon et al. [36].

### 4.5. Dynamic Viscosity and Specific Conductivity

**Figure 5:**
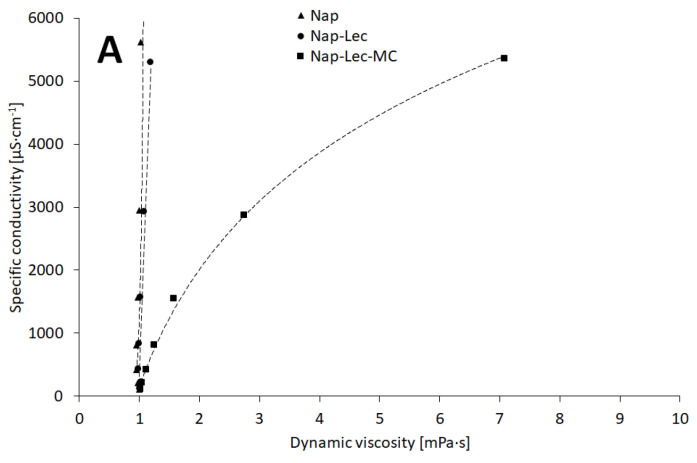

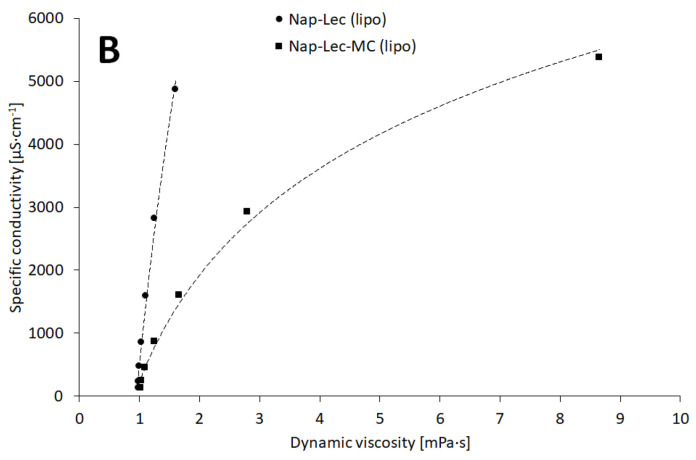
The relation of viscosity and the specific conductivity of evaluated systems in physical mixtures (**A**) and liposomal formulations (**B**). Composition details and abbreviations are outlined in Table 1.

One of the interesting problems of electrical conductivity studies in aqueous systems is the influence of the dynamic viscosity on the mobility of the particles and molecules present within the evaluated mixtures [37,38]. Figure 5 summarizes the values of specific conductivity, as a function of dynamic viscosity. In the selected samples, the active pharmaceutical ingredient—naproxen sodium—is mainly responsible for the observed conductance phenomena. However, other components may add to the recorded conductivity, as was presented above in the Results section. According to the plots, the viscosity, increased by the mean of methylcellulose addition up to 0.25% *w*/*w*, had a very moderate influence on the conductivity of the systems. This enables further studies on electrical conductivity as an important factor, which may be applied in analytical procedures in the pharmaceutical systems of moderate viscosity.

It should be marked that the relation $\kappa_{s}=f\left(\eta \right)$ (Equation (12)), in most cases, adhered most likely to the logarithmic function, as was presented in Table 3, whereas the dependence $\eta =f\left(c_{\%ICS}\right)$ (Equation (11)) followed the pattern of the exponential function. Both the natural logarithmic function and the exponential function confirm the possible co-dependency of conductivity and naproxen sodium concentration in assessed systems; however, further studies should be performed to obtain information on factors useful for practical procedures.

### 4.6. Applicative Importance of the Conductometric Evaluations

To summarize, the naproxen sodium concentration highly influenced the electrical conductivity of the assessed systems, whereas the dynamic viscosity influenced the conductivity results in a rather moderate way. The observed phenomena may be ascribed to the high mobility of sodium ions recruited from naproxen sodium and the relatively low concentrations of methylcellulose in the assessed systems. The assembly of lecithin in the liposomes may be a factor that decreases the specific conductivity of the systems with naproxen sodium. The observed phenomena are of high importance for the development of topically applied drug forms with liposomes immersed into the matrix of hydrophilic polymeric gel. The methylcellulose very moderately influenced the recorded values of specific conductivity. The influence of lecithin, in the form of liposomes or as aqueous dispersion, was intermediate. In contrast, the naproxen sodium concentration was the main factor that added to the elevation of conductivity level, both in the case of the physical mixtures and liposomal preparations. The conductometric method thus may be applied for the evaluation of the drug concentration, as well as in evaluating the naproxen sodium released during the preparation of the acceptor fluid in the course of the drug release or drug dissolution assessments. Two main issues may influence the practical application of the conductometric evaluations of the level of naproxen sodium in the acceptor compartment during the assay, or its level within the preparation. Firstly, the additional components, in the presented case of the lecithin, should be included in the project of a method based on the conductometric evaluations. Secondly, the appropriate results may be obtained in the case of non-ionic polymer, as was the case for methylcellulose. The use of anionic or cationic polymer as the component of the system will require extensive measurements, which could be developed in another work. The measurements of specific conductivity may be further developed for implementation in the simple assays of the level of active pharmaceutical ingredient in preparations proposed for dermatological applications. Also, the aspects of real-time quality control or stability testing in transdermal systems could be covered by in situ or on-line measurements of electric conductivity. However, there is a need for the development of more precise techniques that may enable differentiation between the components of the formulation. Future approaches may include an advanced calculation of the concentrations on the basis of conductivities, using numerous reference samples. However, some implications may hinder this idea, e.g., the need to perform a high number of reference measurements.

## 5. Conclusions

The high differences of specific conductivities were found between liposomal systems with naproxen sodium and without naproxen sodium. The presence of methylcellulose had a moderate impact on the level of the conductivity of liposome formulations with or without the naproxen sodium. The molar conductivities of all evaluated formulations with naproxen sodium, calculated on the basis of naproxen sodium concentration, decreased as the molar concentration increased, according to the pattern close to the logarithmic function, a pattern often observed in molar conductivity assays. Generally, the molar conductivities were lower in the highly diluted samples of non-liposomal preparations with naproxen sodium compared to the liposomal compositions of naproxen sodium. The levels of the limiting molar conductivity of the evaluated systems were very similar in the various assessed systems. The comparison of the results from the specific conductivity assays with the results of the viscosity measurements revealed the low impact of viscosity on the levels of specific conductivity in evaluated systems.

## Figures and Tables

**Table 1:** Composition of evaluated systems.

Formulation Type	Acronym	Naproxen Sodium [mol/L]	Lecithin [%]	Methylcellulose [%]	Water [%]	Evaluated Dilutions of Prepared Formulations							Mark	Line
Physical mixture	Lec	-	1.00	-	99.00	1	1:2	1:4	1:8	1:16	1:32	1:64	○	- . -
Physical mixture	MC *	-	-	0.25	99.75	1	1:2	1:4	1:8	1:16	1:32	1:64	◊	- . -
Physical mixture	Lec-MC *	-	1.00	0.25	98.75	1	1:2	1:4	1:8	1:16	1:32	1:64	□	- . -
Physical mixture	Nap	0.095	-	-	99.00	1	1:2	1:4	1:8	1:16	1:32	1:64	▲	- . -
Physical mixture	Nap-Lec	0.095	1.00	-	98.00	1	1:2	1:4	1:8	1:16	1:32	1:64	●	- . -
Physical mixture	Nap-MC *	0.095	-	0.25	98.75	1	1:2	1:4	1:8	1:16	1:32	1:64	♦	- . -
Physical mixture	Nap-Lec-MC *	0.095	1.00	0.25	97.75	1	1:2	1:4	1:8	1:16	1:32	1:64	■	- . -
Liposomal composition	Lec (lipo)	-	1.00	-	99.00	1	1:2	1:4	1:8	1:16	1:32	1:64	○	-
Liposomal composition	Lec-MC (lipo) *	-	1.00	0.25	98.75	1	1:2	1:4	1:8	1:16	1:32	1:64	□	-
Liposomal composition	Nap-Lec (lipo)	0.095	1.00	-	98.00	1	1:2	1:4	1:8	1:16	1:32	1:64	●	-
Liposomal composition	Nap-Lec-MC(lipo) *	0.095	1.00	0.25	97.75	1	1:2	1:4	1:8	1:16	1:32	1:64	■	-

*—the asterisk indicates the formulations prepared as hydrophilic gels; (lipo)—the “lipo” acronym indicates the formulations with liposomal structures.

**Table 2:** The equation parameters and respective determination coefficients of functions proposed as descriptive for recorded plots: dilution-specific conductivity of the systems, naproxen molar concentrations–molar conductivity of the systems, and dilution-dynamic viscosity of the systems. Composition details and abbreviations are outlined in Table 1.

Relation	Sample	Equation Type of Highest Determination Coefficient	Determination Coefficient [r^2^]
$\kappa_{s}=f\left(c_{\%ICS}\right)$, (9)	Lec	y = 6.0604x^0.832^	0.9996
	MC	y = 0.4302x + 4.0226	0.9980
	Lec-MC	y = 5.3534x^0.8764^	0.9999
	Nap	y = 71.318x^0.9526^	0.9998
	Nap-Lec	y = 77.417x^0.9263^	0.9992
	Nap-MC	y = 67.999x^0.9547^	0.9998
	Nap-Lec-MC	y = 74.66x^0.9339^	0.9997
	Lec (lipo)	y = 19.518x^0.7567^	0.9917
	Lec-MC (lipo)	y = 16.92x^0.8233^	0.9989
	Nap-Lec (lipo)	y = 88.26x^0.8848^	0.9980
	Nap-Lec-MC (lipo)	y = 85.572x^0.9048^	0.9998
$\Lambda_{s}=f\left(c_{mol,\;Nap}\right)$, (10)	Nap	y = −3125ln(x) + 53,064	0.9557
	Nap-Lec	y = −4908ln(x) + 46,418	0.9653
	Nap-MC	y = −2863ln(x) + 51,433	0.9533
	Nap-Lec-MC	y = −4341ln(x) + 47,710	0.9644
	Nap-Lec (lipo)	y = −7794ln(x) + 35,988	0.9400
	Nap-Lec-MC (lipo)	y = −6674ln(x) + 42,021	0.9852
$\eta =f\left(c_{\%ICS}\right)$, (11)	Lec	y = 0.9602e^0.0018x^	0.9289
	MC	y = 0.9541e^0.0171x^	0.9992
	Lec-MC	y = 1.0457e^0.0138x^	0.9943
	Nap	y = 0.9625e^0.0006x^	0.6226
	Nap-Lec	y = 0.9972e^0.0017x^	0.9085
	Nap-Lec-MC	y = 0.9831e^0.0199x^	0.9996
	Lec (lipo)	y = 1.0555e^0.0126x^	0.9977
	Lec-MC (lipo)	y = 0.9201e^0.0234x^	0.9984
	Nap-Lec (lipo)	y = 0.9699e^0.005x^	0.9996
	Nap-Lec-MC (lipo)	y = 0.9647e^0.0218x^	0.9998

**Table 3:** The equation parameters and respective determination coefficients of functions proposed as descriptive for recorded plots: viscosity–molar conductivity of the systems (Equation (12)). Composition details and abbreviations are outlined in Table 1.

Relation	Sample	Equation Type of Highest Determination Coefficient	Determination Coefficient [r^2^]
$\kappa_{s}=f\left(\eta \right)$, (12)	Lec	y = 1435.4x − 1360.4	0.9200
	MC	y = 25.126ln(x) + 5.2273	0.9950
	Lec-MC	y = 218.81ln(x) − 0.5934	0.9868
	Nap	y = 7 × 10^−8^e^23.687x^	0.7541
	Nap-Lec	y = 26013x − 25611	0.8792
	Nap-Lec-MC	y = 2677.3ln(x) + 156.28	0.9976
	Lec (lipo)	y = 446.11ln(x) + 30.135	0.9784
	Lec-MC (lipo)	y = 297.03ln(x) + 66.775	0.9788
	Nap-Lec (lipo)	y = 9565.5ln(x) + 493.86	0.9923
	Nap-Lec-MC (lipo)	y = 2438.9ln(x) + 236.7	0.9952

## Data Availability

The original contributions presented in the study are included in the article, further inquiries can be directed to the corresponding author.
